# Analysis of the dermatophyte *Trichophyton rubrum *expressed sequence tags

**DOI:** 10.1186/1471-2164-7-255

**Published:** 2006-10-11

**Authors:** Lingling Wang, Li Ma, Wenchuan Leng, Tao Liu, Lu Yu, Jian Yang, Li Yang, Wenliang Zhang, Qian Zhang, Jie Dong, Ying Xue, Yafang Zhu, Xingye Xu, Zhe Wan, Guohui Ding, Fudong Yu, Kang Tu, Yixue Li, Ruoyu Li, Yan Shen, Qi Jin

**Affiliations:** 1 State Key Lab for Molecular Virology and Genetic Engineering, Beijing 100176, China; 2 Research Centre for Medical Mycology, Beijing 100034, China; 3 Bioinformatics Center, Shanghai Institutes for Biological Sciences, Chinese Academy of Sciences, Shanghai 200031, China; 4 Chinese National Human Genome Center, Beijing, Beijing 100176, China; 5 The Institute of Pathogen Microbiology, Chinese Academy of Medical Science, Beijing 100730, China

## Abstract

**Background:**

Dermatophytes are the primary causative agent of dermatophytoses, a disease that affects billions of individuals worldwide. *Trichophyton rubrum *is the most common of the superficial fungi. Although *T. rubrum *is a recognized pathogen for humans, little is known about how its transcriptional pattern is related to development of the fungus and establishment of disease. It is therefore necessary to identify genes whose expression is relevant to growth, metabolism and virulence of *T. rubrum*.

**Results:**

We generated 10 cDNA libraries covering nearly the entire growth phase and used them to isolate 11,085 unique expressed sequence tags (ESTs), including 3,816 contigs and 7,269 singletons. Comparisons with the GenBank non-redundant (NR) protein database revealed putative functions or matched homologs from other organisms for 7,764 (70%) of the ESTs. The remaining 3,321 (30%) of ESTs were only weakly similar or not similar to known sequences, suggesting that these ESTs represent novel genes.

**Conclusion:**

The present data provide a comprehensive view of fungal physiological processes including metabolism, sexual and asexual growth cycles, signal transduction and pathogenic mechanisms.

## Background

Dermatophytes, consisting of organisms in the *Trichophyton, Epidermophyton*, and *Microsporum *genera, are the primary etiologic pathogens of various dermatophytoses, such as tinea capitis, tinea corporis, tinea inguinalis, tinea manus, tinea unguium and tinea pedis. These infections are widespread and increasing in prevalence on a global scale. Indeed, in some geographic regions, dermatophyte infection is now considered a major public health concern.

Unlike other fungi, dermatophytes can cause infections in healthy, immune-competent individuals. Estimates suggest that 30 to 70% of adults are asymptomatic carriers of these fungi [1]. *T. rubrum *is the most common superficial fungus, accounting for at least 60% of all superficial fungal infections in humans. This organism may remain viable in the environment for over six months, thus accounting for widespread infections. Transmission occurs most often from person to person [2], for example, by shedding of infected skin cells and hair and by direct body contact [3].

Diagnosis of dermatophyte infections relies on clinical presentation, requiring successful isolation and culture and microscopy. It will take 2 to 4 weeks to culture and pleomorphic growth can lead to misidentification. Sometimes the morphological phenotype is not very stable. Rapid diagnostic tests using current molecular methodologies have been slow to develop for the dermatophytes [4].

Furthermore, despite the availability of new systemic antifungal therapies, nail infections are particularly difficult to eradicate, presenting a 25 to 40% recurrence rate [5]. Another increasing problem in clinical treatments is growing resistance to antifungal drugs. In the past decade, more and more cases of azole- and terbinafine-resistant fungal infections have been reported [6-8]. Ryder *et al *[9,10] characterized at the molecular level the cause of the resistant phenotype of some clinical isolates, considering the resistance in some *T. rubrum *clinical isolates to terbinafine appears to be due to a single amino acid substitution in the gene squalene epoxidase.

*T. rubrum *is anthropophilic, specialized to infect humans, but rare animal infections have been reported. It is urease negative and vitamins independent. On the Bromcresol purple-(BCP)-milk solids glucose media, it shows restricted growth, no indicator color change for 10 to 14 days, then profuse growth with indicator change to purple (alkaline). Its teleomorph has not yet been found. Arthroconidia and mycelial fragments are the infectious agents. Excreted extracellular proteinase, alkaline serine proteinase, plays a role in dermatophytes growth and multiplication as well as in the inflammatory reaction [2,11-13]. Thus, they are considered to be critical virulence factors for dermatophytes. There are many reports of the isolation and characterization of one or two proteases from an individual species of dermatophyte [14-18]. In *T. rubrum*, Some keratinases have been isolated and a subtilisin gene family were identified at the genetic level [19-22]. Jousson *et al *isolated a five-member secreted metalloproteases (MEP) family from genomic libraries of *T. rubrum, T. mentagrophytes *and *M. canis*. Further phylogenetic analysis revealed that the metalloproteases secreted by the three species in vitro are encoded by orthologous genes, strongly suggesting that the multiplication of an ancestral metalloprotease gene occurred prior to the dermatophyte species divergence [23]. However, only a small number of proteases from dermatophytes have been characterized at the genetic level.

Taken together, the lack of effective diagnostic and treatment strategies, the large number of individuals that experience dermatophyte infections, and the economic consequences highlight deficiencies in the research efforts aimed at understanding dermatophyte. *T. rubrum *is the most common superficial fungus, inducing dermatophytoses in various parts of the human skin, and can also cause deeper infections such as kerions, abscesses and granulomas. Research examining the pathogenesis of *T. rubrum *in numerous skin infections is needed in order to develop novel therapeutic agents and identify potential drug targets.

In the present study, we constructed various *T. rubrum *cDNA libraries and obtained 11,085 unique expressed sequence tags (ESTs). By analyzing these ESTs, we were able to further elucidate the physiological activities in metabolism, signal transduction, sexual life cycle, pathogenesis and cell wall composition of *T. rubrum*. These sequences provide an unparalleled resource for the future understanding of this remarkable fungus. Furthermore *T. rubrum *represents an attractive model species with which to study dermatophytes and other pathogenic filamentous fungi.

## Results and discussion

### Expressed sequence tags sequence determination

EST sequences were produced from 10 different cDNA libraries. After trimming for the vector and poor quality segments, a total of 34,670 sequences were identified and 11,085 unique ESTs were isolated. Isolated ESTs included 3,816 contigs and 7,269 singletons. All the constructed libraries, culture conditions and sequencing statistics employed in this study are summarized in table 1. The average sequence length of these unique ESTs was 586 nucleotides (nt). Approximately 8,639 (78%) of the unique ESTs were longer than 400 nt.

In the synthesis of the cDNA first strand, incomplete cDNA synthesis and/or 5' truncation of mRNA transcripts could lead to an overestimation of the gene number identified from our analysis. Accuracy of the estimation absolutely depends upon the quality of the library. Among the 7,764 matched ESTs, 1,439 could be matched to reference genes if the subjected starting site was set to be the first coding amino acid; meanwhile 2,325 matching genes could be found if the subjected starting site was allowed to be any of the first 10 amino acids. These data confirm that the quality of the libraries is reliable.

### Comparison to Non-redundant and Clusters of Orthologous Groups databases

Identified ESTs were compared with the GenBank non-redundant (NR) protein database. This comparison enabled us to assign putative functions to or find homologs from other organisms for 7,764 (70%) of the ESTs. The remaining 3,321 (30%) ESTs were only weakly similar or not similar to known sequences (E ≥ 1E-05), suggesting that they represent novel genes.

The comparative results for the ESTs with respect to the Clusters of Orthologous Groups (COGs) database, and its eukaryotic counterpart termed the KOGs database, are summarized in table 2. The ESTs identified herein contained a broad range of genes, predominantly encoding putative proteins involved in primary metabolism, gene expression, post-translation processes and cell structure. A significant proportion of the identified ESTs were matched to genes involved in transcription and signal transduction, suggesting that *T. rubrum *has an elaborate regulation system.

The COG collection currently consists of 138,458 proteins, which form 4873 COGs and comprise 75% of the 185,505 (predicted) proteins encoded in 66 genomes of unicellular organisms, including 3 Eukaryota, *Saccharomyces cerevisiae, Schizosaccharomyces pombe *and *Encephalitozoon cuniculi*. The eukaryotic orthologous groups (KOGs) include proteins from 7 eukaryotic genomes: three animals (*Caenorhabditis elegans, Drosophila melanogaster *and *Homo sapiens*), one plant, *Arabidopsis thaliana*, two fungi (*Saccharomyces cerevisiae *and *Schizosaccharomyces pombe*), and the intracellular microsporidian parasite *Encephalitozoon cuniculi*. The current KOG set consists of 4852 clusters of orthologs, which include 59,838 proteins, or ~54% of the analyzed eukaryotic 110,655 gene products [24]. Because the RNA processing in eukaryotic cells is more elaborated, and eukaryotic cells have some structures that prokaryotic cells lack, for example, nuclear and mitochondria, more genes involved in RNA processing and modification [A], chromatin structure and dynamics [B], cell cycle control, cell division, chromosome partitioning [D], nuclear structure [Y], cytoskeleton [Z] and intracellular trafficking, secretion, and vesicular transport [U] were identified in KOGs than COGs. The difference between gene number of class energy production and conversion [C] was mainly due to the mitochondrial related genes. On the contrary, more genes involved in cell motility [N], mainly encoding secretory pathway and cell motivity related proteins, were identified in COGs than KOGs. Meanwhile, the COG/KOG result was also affected by the subtle difference in functional classification to function similar proteins, especially transporters.

### Comparison of ESTs to those already available in GenBank

To date 3,749 *T. rubrum *ESTs have been released to GenBank by other research groups, most of which (3,711) were obtained in condition mimicking virulence by Zaugg and Monod *et al*. When we compared these 3,749 previously reported ESTs to the NR database and to our EST pool, we found that 3,112 of the previously described ESTs in GenBank had matching homologs in other organisms in the NR database (E <1E-05) and that 637 ESTs were only weakly similar or did not match known sequences. Among the 3,749 ESTs identified previously, 2,738 ESTs matched 1,325 unique ESTs in our data (E <1E-05, identity ranges from 72% to 100%), suggesting these data be somewhat redundant. The remaining 1,011 did not have matching homologs in our EST database, including 263 ESTs which had no homology whatsoever in the NR database. Meanwhile, 748 of the 1,011 ESTs obtained only in their research had some homology with sequences in the NR database (E <1E-05), while 620 ESTs appeared to be hypothetical proteins, predicted proteins or unnamed protein. Some secreted proteinases that were identified by Monod *et al*. were not identified in our study (i.e. subtilisin-like protease SUB4, dipeptidyl-peptidase IV, leucine aminopeptidase 1, leucine aminopeptidase 2 and a putative secreted metalloprotease 4), suggesting that the condition in their research may be a more suitable environment to induce expression of these proteins. The proteinases subtilisin-like protease SUB1 and subtilisin-like protease SUB5 were expressed not only in the condition they mimicked but also in YPD media (see Additional file 1). By comparison with previous released ESTs, our data supply the information about gene expression in different conditions, advancing the current knowledge of *T. rubrum *transcriptome.

### Metabolism and secondary metabolism

#### Metabolic overview

A large percentage of *T. rubrum *genes were annotated in glycolysis and oxidative phosphorylation systems, as expected from what is known from other aerobic filamentous fungi. Genes corresponding to the citrate cycle enzymes and to components of complexes I (NADH-CoQ reductase), II (Succinate-CoQ reductase), III (CoQ-Cytochrome C reductase), and IV (Cytochrome C oxidase) were identified. The presence of these genes reflects the fungus' ability to perform complete aerobic pyruvate degradation and oxidative phosphorylation.

The pathways involved in metabolizing mono- and disaccharides, such as glucose, fructose, mannose, and sucrose, as well as polysaccharide starch, were best represented in the identified ESTs. There were also a large number of membrane transporters for saccharides such as xylose and fucose. Among the identified ESTs, all aminoyl-tRNA synthases have been previously described with the exception of cyseine-tRNA synthase, of which only one contig was found to exhibit weak homology (C3495-Contig1, E value 0.00008). Comparison of our ESTs data to KEGG revealed that these proteins were probably involved in many amino acid metabolism pathways, including glutamate metabolism, alanine and aspartate metabolism, glycine, serine and threonine metabolism, methionine metabolism, cysteine metabolism, valine, leucine and isoleucine biosynthesis, lysine biosynthesis, arginine and proline metabolism, histidine metabolism, phenylalanine, tyrosine and tryptophan biosynthesis. Analysis of these pathways indicated that *T. rubrum *could itself synthesize lysine. It could synthesize some amino acids via amino acid synthetase including glutamate, glutamine, asparagine, cysteine, tryptophan and threonine, and it can also synthesize some other amino acids via amino transfer reactions. The primary lipids in *T. rubrum *are sterol and phospholipid. Our analysis of the metabolic pathways in *T. rubrum *revealed that it can also synthesize co-enzymes such as riboflavin, nicotinate, nicotinamide, coenzyme A, ubiquinone, and folate. The genes probably involved in the synthesis of thiamine included: NMT1 protein, THI4 protein, ThiC protein and a putative ThiG protein. Because *T. rubrum*'s vitamin independence serves to separate it from species such as *Trichophyton violaceum *that have vitamin requirements, identification of the ESTs involved in vitamin synthesis is of great significance for molecular diagnosis. Some ESTs probably involved in metabolism are included in additional files (see Additional file 2).

#### Secondary metabolism

Secondary metabolites are a remarkably diverse class of cellular products that often exhibit taxonomic specificity. Secondary metabolites are generally considered "nonessential" for organismal growth in culture. In addition to the metabolic pathways mentioned above, there are also several secondary metabolic pathways present in *T. rubrum*. It has long been known that dermatophytes can survive in toxic environments, presumably through the production of biological tools to resist these toxins. Indeed dermatophytes have even been isolated from sewer water and polluted soil containing organic compounds such as aromatic compounds. The pathways implicated by the EST analysis are consistent with *T. rubrum*'s capability to enzymatically degrade various toxins such as phenanthrene, dibenzofuran, ethylbenzene, styrene, fluorene, and 1,1,1-Trichloro-2,2-Bis-(4'-Chlorophenyl)Ethane(DDT) (see Additional file 3).

Similar to other pathogenic filamentous fungi, such as *Magnaorthe grisea *[25] and *Aspergillus fumigatus *[26], several important secondary metabolic products, were also found in *T. rubrum*; these included non-ribosomal peptide synthases, polyketide synthases, two putative dimethylallyl tryptophan synthases, a putative arsenate reductase and a hydrophobin. These gene products may be related to *T. rubrum *growth and pathogenicity. Polyketides (derived from polyketones) are a class of secondary metabolites produced by most organisms, but they have been most extensively examined in bacteria and fungi. In fungi, numerous functions have been proposed for polyketides, including the production of toxins [27,28] and spore pigments [29,30]. Although it is well known that *T. rubrum *can produce pigments, the relationship between secondary metabolic pathways and pigments production remains unresolved. The various identified ESTs involved in secondary metabolism are listed in additional files (see Additional file 3).

Interestingly, *T. rubrum *harbors a putative sterigmatocystin biosynthesis monooxygenase StcW (C1113-Contig1), and a probable sterigmatocystin biosynthesis P450 monooxygenase STCL (Cytochrome P450 60B, EST000637, [GenBank:DW406216]), suggesting that it very likely may produce sterigmatocystin. However, confirmation of this possibility will require further investigation. Sterigmatocystin is of particular significance in evaluating the toxicity of *T. rubrum *products because prior evidence indicates that it is probably toxic to the human liver.

Although it is suspected that *Trichophyton mentagrophytes *can produce a penicillin-like substance [31], we did not find any evidence suggesting that *T. rubrum *generates antibiotic-like by-products.

### Signal transduction

We also identified a variety of signal transduction systems in *T. rubrum*, such as MAPK, cAMP-dependent pathways, G-protein pathways, Ras pathways, and a large number of serine/threonine protein kinase/phosphatases and signal histidine kinases. Although the first four systems are generally conserved among fungal and mammalian species, the numbers and functions of signal transduction of histidine kinases vary between fungi. For example, in *S. cerevisiae *there exists only one, but in *Neurospora crassa *[32] there are 11 different histidine kinases. Indeed, there are many differences in the numbers and types of histidine kinases between filamentous fungi and yeast. Some experimental results [33-36] obtained with *Candida albicans *and *Aspergillus fumigatus *suggest that histidine kinases are related to fungal pathogenesis. In *T. rubrum *many ESTs are homologous to histidine kinases, some of which may be related to osmotic and nutrient responses. Several identified histidine kinases contain PAS/PAC domains, suggesting that they are involved in oxygen and light responses.

In addition to histidine kinase-related ESTs, we also identified Calcium-Calmodulin homologues and related proteins, suggesting that Ca-CaM pathways may be utilized by *T. rubrum*, similar to the model organism *Neurospora crassa*. Commonly, Ca^2+ ^release from internal stores is mediated by the second messengers inositol-1, 4, 5-trisphosphate (InsP3) and cADP ribose, or by Ca^2+^-induced Ca^2+ ^release [37]. Although InsP3 is present within *Neurospora *hyphae [38] and *T. rubrum, Neurospora *lack recognizable InsP3 receptors, ADP ribosyl cyclase and ryanodine receptor proteins, which are principal components of Ca^2+ ^release mechanisms in plant and animal cells. These observations raise the question of whether there may be other second messenger systems that are responsible for Ca^2+ ^release from internal stores that remain to be discovered in filamentous fungi. InsP3 receptors, ADP ribosyl cyclase or ryanodine receptor proteins were also not found in our EST data (the existence of these genes can only be determined after the whole genome is sequenced). Thus the mechanism of *T. rubrum *Ca-CaM pathways remains to be determined. The various identified ESTs involved in signal transduction are listed in additional files (see Additional file 4).

### Sexual and asexual development

Although the sexual life cycle has been described for *Trichophyton mentagrophytes, Trichophyton ajelloi *and *Microsporum canis *[2,39], which are all heterothallic species, the sexual cycle of *T. rubrum *remains to be elucidated. We compared sexual-cycle related genes with those in other fungi, and our findings suggest that *T. rubrum *may be capable of sexual reproduction.

Sexual reproduction in ascomycete filamentous fungi is governed, in part, by two different mating-type genes that establish sexual compatibility: one gene encodes a protein with a high mobility group (HMG) domain, and the other encodes a protein with an alpha box domain (MAT). Such MAT containing genes are termed alpha mating-type genes. Homothallic fungi typically possess both mating-type genes and are self-fertilizing. Heterothallic fungi possess only one mating-type gene and require a partner with a different complementary mating-type gene. Although the MAT locus has not yet been found in *T. rubrum*, HMG is present (4 ESTs: EST000702, [GenBank:DW406281]; EST001404, [GenBank:DW406983]; EST004330, [GenBank:DW680849]; EST004146, [GenBank:DW680665]), and a large number of meiosis-related genes were identified in the ESTs, many of which were also found in *Aspergillus niduluns *and/or *A. fumigatus*. An analysis of 215 genes implicated in the fungal mating process, pheromone response, meiosis and fruiting body development revealed that many genes present in *A. nidulans *[26,40,41] are also present in *T. rubrum*, including several genes for which the only known function is related to sexual reproduction (see Additional file 5). These results suggest that *T. rubrum *may also possess sexual cycles. However more in-depth exploration will be required in order to determine whether they indeed possess sexual cycles.

The current research on the fungal asexual development cycle primarily involves studies of *Neurospora crassa *and *Aspergillus niduluns*, and the literature contains two distinct models. Our comparison of the identified *T. rubrum *ESTs with the NR protein database, revealed a key enzyme (FlbD, EST023258, [GenBank:DW699777], E value 5E-52) that is present in *A. niduluns *[41], but no key enzymes present in *Neurospora crassa*. Because in *A. nidulans *four proteins (FlbC, BrlA, AbaA and WetA) besides FlbD are also required in macroconidiation pathway, it is still unclear whether *T. rubrum *is more similar to *A. niduluns *or *N. crassa *with regards to asexual development.

### Extracellular proteinases

The most evident dermatophyte feature is the ability to digest keratin. Dermatophytes can degrade human and other animal keratin protein and utilize it. This represents the pathogenic feature that differentiates dermatophytes from other fungi. The secreted proteinases of dermatophytes play an important role in the process of infection and are thus considered the primary virulent factors. However, only a few proteinase sequences have been identified thus far in *T. rubrum *[19-22,42-45]. In our data, secreted proteins were firstly analyzed by the NR comparison result. To the putative secreted protein identified in NR database, all of the corresponding ESTs were further subjected to SignalP prediction analysis. As to the ESTs listed in the Additional file 6, 48 of them were predicted to contain a signal peptide. Among the *T. rubrum *ESTs, identified secretory systems include type I, II, III and V, with type II, III being the most heavily represented. We also identified a large number of putative secretion related pathways and putative secreted proteinase, including some serine proteinases, aspartic protease, alkaline proteinase, peptidases and metalloprotease. (see Additional file 6). Among the serine proteinases, 10 unique ESTs were homologous to the known dermatophyte subtilisin-like serine protease family members SUB1, SUB5, and SUB6 [22]; meanwhile no identified ESTs were homologous to SUB2, SUB3, SUB4, or SUB7. The identity of the 6 serine protease ESTs with highest homology to *T. rubrum *serine proteinases varied from 80% to 93%, suggesting that the genes may be selectively spliced, and/or the family may have other members, and/or that these genes have many copies in the genome.

Secreted metalloproteases are thought to be associated with lesion extension [15]. Recent reports [46] have identified a nitrogen regulating factor response region located upstream of the *Microsporum canis *MEP1 gene, consistent with the finding that, at the beginning of infection, there are only a few urea molecules, amino acids and glucose molecules available as a nutrient resource in sweat. Aspartic protease is an acid proteinase whereas secreted metal proteinase and subfamily members are neutral or basic proteinases. The presence of these proteinases supports the finding that initially *T. rubrum *can grow in either acidic or basic pH environments.

These proteinase and peptidase findings suggest that they may also be related to amino acid transportation. During *T. rubrum *infection, extracellular proteins are hydrolyzed into peptides by secreted proteinases then are further degraded into amino acids or dipeptides by peptidases and finally transported into cells. The identification of these proteinases and peptidases increases our understanding of the pathogenic mechanism underlying *T. rubrum *infection.

These secreted proteinases can not only degrade proteins such as keratin, elastin and collagen to supply nutrients to the fungi, but can also induce delayed-type hypersensitivity (such as with the SUB family and Tri r4) [1,22]. However, although the *T. rubrum *allergen Tri r4 EST shares 98% homology with that in *Trichophyton mentagrophytes*, it is clear that the inflammation mediated by *T. rubrum *is not as severe as that induced by *T. mentagrophytes *. This is probably due, at least in part, to the presence of multiple inflammatory factors in *T. mentagrophytes*. When comparing the putative virulence factors present in *T. rubrum *with those in *A. fumigatus *[26] we also observed that many known virulence factors in *A. fumigatus *were not present in the identified *T. rubrum *ESTs. Likewise, many putative *T. rubrum *virulence factors were not found or were divergent in *A. fumigatus*. For example, the *mep*1 gene exists in both *A. fumigatus *(Afu8g07080) and dermatophytes, but the two sequences were highly divergent (E value 5e-26).

### Cell wall

The cell wall is a structure that humans lack but that fungi have. Therefore, the cell wall represents an ideal target for novel anti-fungal drugs. Many cell wall-related proteins were found among the presently identified ESTs, including chitin synthesis, chitinase, β1,3-glucan synthase, β1,6-glucan synthase, and 1,4-alpha-glucan branching enzyme. By analyzing the pathways involved in cell wall synthesis, it can be inferred that *T. rubrum *is probably capable of synthesizing peptidoglycan, the primary component of gram-positive bacteria. The principle enzymes involved in the peptidoglycan synthesis pathway UDP-N-acetylmuramoylalanyl-D-glutamyl-2, 6-diaminopimelate-D-alanyl-D-alanine ligase (EST020893, [GenBank:DW697412], GO:0008766) and phospho-N-acetylmuramoyl-pentapeptide-transferase (EST009048, [GenBank:DW685567], EC:2.7.8.13) were identified in our EST data. But it is still to be determined by experimental confirmation. We also identified some putative proteins that are likely to be involved in sterol synthesis, which is a primary target for clinically-available drugs. The components of the identified ESTs that are involved in cell wall synthesis are listed in table 3.

## Conclusion

The estimated *T. rubrum *genome size is at least 22.05 Mb [47]. And we estimate that a significant proportion of the gene content of *T. rubrum *is represented in this collection of sequences. Although ESTs can only represent genes that are actively expressed in particular phases, they complement the value of genomic sequencing through the functional identification of novel genes and provide information about gene structure and expression patterns [48-53]. Thirty percent of the 11,085 contigs and singletons identified in our analysis represent unique genes. Our Blast searches revealed that 33% of the 11,085 unique sequences possess matches in the yeast genome (<e-5), suggesting that there are clear distinctions (~67%) between yeast and filamentous fungi. These findings highlight the need for additional research on filamentous fungi.

In summary, although superficial fungi are the primary human pathogenic fungi, our knowledge about these organisms remains limited. *T. rubrum *represents an ideal model for the study of superficial fungi; our work in identifying ESTs in *T. rubrum *cDNA libraries will facilitate a greater understanding of the molecular mechanisms underlying its growth, metabolism, pathogenesis and drug resistance. In addition, our work may aid in the identification of novel effective drug targets and anti-fungal agents.

## Methods

### Preparation of strains and materials

*T. rubrum *(strain BMU01672) and medium have been described previously [54].

### Preparation of cell cultures

#### Preparation of hyphae cultures

A few hyphae of *T. rubrum *were inoculated on potato glucose agar and incubated at 27.5°C for 2~3 weeks. In order to get cDNAs covering the entire growth phases, the hyphae were inoculated in liquid YPG medium (10 g/L yeast extract, 20 g/L peptone, 10 g/L D-glucose) and incubated in a 27.5°C bath shaker for 7, 10, 14, 15, 16, 20, 22, 26, 28, 34 and 36 d, respectively. Combine the mycelium from (7,10, 15, 20, 28) (7, 10, 16, 22, 26, 28), (16, 20, 22), (26, 28), and (34, 36) days, thus get 8 samples (or mixtures) from 7, 10, 14, (7, 10, 15, 20, 28), (7, 10, 16, 22, 26, 28), (16, 20, 22), (26, 28),(34, 36) day. All the samples or mixtures were used to construct unnormalized cDNA libraries except the mixture (7, 10, 15, 20, 28), which was used to prepare the tester cDNA of a subtracted cDNA library. A large number of sequenced plasmids in 7, 10, and 14 days libraries were in vitro transcripted (Riboprobe^® ^in vitro Transcription Systems, Promega) and the RNA products were further reverse transcripted into the driver cDNA (Clontech PCR-Selected cDNA Subtraction Kit). The fungal samples (or mixtures) were centrifuged and the supernatant were discarded. The pelleted mycelium were washed twice with PBS.

#### Preparation of spore cultures

A few hyphae of *T. rubrum *were inoculated on potato glucose agar and incubated at 28°C for 2~3 weeks. The spores were then washed by liquid YPG medium and filtered by cell filter to get rid of the hyphae. Part of the spores obtained were incubated in YPG medium at 28°C for 12 hours. Finally we got two samples, spores and incubated spores, respectively.

### Isolation and purification of total RNA and mRNA

Total RNA and mRNA were isolated and purified as described previously [54].

### Construction of the cDNA library

#### Construction of the unnormalized cDNA library

The 9 cDNA libraries (7 representing mycelium and 2 representing spores) were constructed following the protocols of the SUPERSCRIPT™ Plasmid System with GATEWAY™ Technology for cDNA Synthesis and Cloning (Invitrogen).

#### Construction of the subtracted cDNA library

The subtracted library was constructed following the protocols of the Clontech PCR-Selected cDNA Subtraction Kit. According to the instruction of the Kit, driver cDNA will be subtracted from the tester cDNA therefore a normalized library is constructed.

### Isolation and purification of plasmids, and sequencing of clones

The cDNA plasmids were isolated as described previously [54]. Sequencing was performed with a generic T7 primer located 5' upstream of the inserted segments, following the protocol of the PRISM Big Dye Terminator Kit on an ABI3700 or MEGABACE automated sequencer.

### Bioinformatic analysis

#### Sequence data processing, EST clustering and re-assembly

The processing method has been described previously [54].

#### EST analysis and construction of the metabolic pathway

The clustered EST consensus sequences were assigned with potential functions through homologous comparisons by BLASTX searches of the GenBank non-redundant (NR) protein database (07/2005), Gene Ontology (GO, 11/2005) and Yeast Genome Database (YGD, 11/2005). ESTs were further classified according to the NCBI Clusters of Orthologous Groups of Proteins database (COGs, 06/2005) and the Eukaryotic Orthologous Groups (KOGs, 06/2005). The metabolic pathways of *T. rubrum *were partially reconstructed by searching for known pathway homologs found in the Kyoto Encyclopedia of Genes and Genomes database (KEGG, 11/2004). Secreted proteins were analyzed by comparison to NR. All the threshold cutoff were E<1E-05. As to the putative secreted protein identified in NR database, all of the corresponding ESTs were further subjected to SignalP prediction analysis.

#### Accession numbers

The sequences have been submitted to GenBank (accession numbers from [GenBank:DW405580] to [GenBank:DW407270] and from [GenBank:DW678211] to [GenBank:DW711189]). All the information about GenBank numbers, contigs assembly, and ESTs annotation could be obtained form our *Trichophyton rubrum *database [55].

## Authors' contributions

LW performed construction of the cDNA gene libraries, clone isolation, plasmids sequencing, data analysis and drafted the manuscript. LM, WL performed construction of the cDNA gene libraries, clone isolation, and plasmids sequencing. TL, LY participated in the construction of the cDNA libraries and the isolation of clones. JY carried out the bioinformatics analysis and constructed the *T. rubrum *database. LY, WZ, QZ, JD, YX, YZ and XX participated in the library construction, clone isolation and plasmids sequencing. ZW and RL were responsible for the strain identification, culture and growth conditions design. GD, FY, KT and YL participated in bioinformatics analysis. QJ designed the project, supervised the research and revised the manuscript. SY cooperated with QJ and implemented the project and supervise the research in the center of Chinese National Human Genome Center. All authors read and approved the final manuscript.

## Supplementary Material

Additional File 1 ESTs Comparison. Click here for file

Additional File 2 metabolism overview. Click here for file

Additional File 3 secondary metabolism. Click here for file

Additional File 4 signal transduction. Click here for file

Additional File 5 Sexual and asexual development. Click here for file

Additional File 6 secreted proteins. Click here for file

## Figures and Tables

**Table 1:** Libraries, culture times, and sequencing statistics for ESTs generated in this study

**ID**	**Library Description**	**EST No.**	**GenBank Acc No.**	**Total**	**Reads in contigs**	**Reads in singletons**
0	Spores incubated in YPG medium for 12 hours	EST001692–EST004492	DW678211–DW681011	2801	1865	936
1	10 days	EST004493–EST010729	DW681012–DW687248	6237	5208	1029
2	14 days	EST010730–EST015367	DW687249–DW691886	4638	3946	692
3	7 days	EST015368–EST017321	DW691887–DW693840	1954	1621	333
4	7, 10, 15, 20 and 28 days culture, Subtracted library	EST017322–EST019168	DW693841–DW695687	1847	1645	202
5	7,10,16,22,26 and 28 days	EST019169–EST020693	DW695688–DW697212	1525	1207	318
6	16,20 and 22 days	EST020694–EST023912	DW697213–DW700431	3219	2267	952
7	26 and 28 days	EST023913–EST030936	DW700432–DW707455	7024	5562	1462
8	34 and 36 days	EST030937–EST034670	DW707456–DW711189	3734	2912	822
9	Spores	EST000001–EST001691	DW405580–DW407270	1691	1168	523

**Table 2:** The comparative results of ESTs to COGs and KOGs

	**Functional Classification**	**Numbers in COG**	**Percents in COG**	**Numbers in KOG**	**Percents in KOG**
	**Information storage and processing**				

J	Translation, ribosomal structure and biogenesis	413	3.73%	408	3.68%
A	RNA processing and modification	21	0.19%	165	1.49%
K	Transcription	378	3.41%	131	1.18%
L	DNA replication, recombination and repair	213	1.92%	85	0.77%
B	chromatin structure and dynamics	32	0.29%	48	0.43%

	**Cellular Processes and Signaling**				

D	Cell cycle control, cell division, chromosome partitioning	49	0.44%	86	0.78%
Y	Nuclear structure	3	0.03%	19	0.17%
V	Defense mechanisms	70	0.63%	17	0.15%
T	Signal transduction mechanisms	240	2.17%	209	1.89%
M	Cell wall/membrane/envelope biogenesis	152	1.37%	37	0.33%
N	Cell motility	64	0.58%	1	0.01%
Z	Cytoskeleton	45	0.41%	95	0.86%
W	Extracellular structures			4	0.04%
U	Intracellular trafficking, secretion, and vesicular transport	153	1.38%	244	2.20%
O	Posttranslational modification, protein turnover, chaperones	345	3.11%	424	3.82%

	**Metabolism**				

C	Energy production and conversion	365	3.29%	315	2.84%
G	Carbohydrate transport and metabolism	409	3.69%	188	1.70%
E	Amino acid transport and metabolism	669	6.04%	343	3.09%
F	Nucleotide transport and metabolism	120	1.08%	110	0.99%
H	Coenzyme transport and metabolism	179	1.61%	118	1.06%
I	Lipid transport and metabolism	256	2.31%	219	1.98%
P	Inorganic ion transport and metabolism	402	3.63%	118	1.06%
Q	Secondary metabolites biosynthesis, transport and catabolism	188	1.70%	131	1.18%

	**Poorly characterized**				

R	General function prediction only	844	7.61%	530	4.78%
S	Function unknown	219	1.98%	154	1.39%

	**Total**	11085			

**Table 3:** Cell-wall related genes in *Trichophyton rubrum *expressed sequence tags

**Contig/EST Number**	**GenBank Acc No.**	**E_value**	**Product**
C0133-Contig1		9.00E-08	chitin binding protein [*Magnaporthe grisea*]
C0210-Contig1		1.00E-31	1,3-beta-glucan biosynthesis protein, putative [*Aspergillus fumigatus *Af293]
C0792-Contig1		4.00E-80	chitin synthase class VI [*Coccidioides immitis*]
C0963-Contig1		1.00E-117	glucan synthase [*Paracoccidioides brasiliensis*]
C0974-Contig1		2.00E-67	chitin biosynthesis protein (Chs5), putative [*Aspergillus fumigatus *Af293]
C1036-Contig1		1.00E-08	beta-1,6-glucan boisynthesis protein (Knh1), putative [*Aspergillus fumigatus *Af293]
C1088-Contig1		6.00E-19	vegetative cell wall protein gp1 [*Aspergillus fumigatus *Af293]
C1127-Contig1		2.00E-95	class V chitinase, putative [*Aspergillus fumigatus *Af293]
C1192-Contig1		8.00E-28	chitin biosynthesis protein (Chs7), putative [*Aspergillus fumigatus *Af293]
C1439-Contig1		1.00E-41	chitin biosynthesis protein (Chs7), putative [*Aspergillus fumigatus *Af293]
C1503-Contig1		8.00E-72	cell wall glucanase, putative [*Aspergillus fumigatus *Af293]
C1548-Contig1		4.00E-29	beta-1,3-glucanosyltransferase 3 [*Paracoccidioides brasiliensis*]
C1553-Contig1		1.00E-73	chitinase [*Ajellomyces capsulatus*]
C1612-Contig1		5.00E-87	beta (1–3) glucanosyltransferase 2 [*Paracoccidioides brasiliensis*]
C1924-Contig1		7.00E-66	cell wall glucanase (Scw11), putative [*Aspergillus fumigatus *Af293]
C1945-Contig1		1.00E-124	1,4-alpha-glucan branching enzyme [*Aspergillus fumigatus *Af293]
C1994-Contig1		1.00E-103	beta-1,6 glucan synthetase (Kre6), putative [*Aspergillus fumigatus *Af293]
C2050-Contig1		4.00E-77	beta-1,3-glucanosyltransferase 3 [*Paracoccidioides brasiliensis*]
C2307-Contig1		1.00E-106	COG1215: Glycosyltransferases, probably involved in cell wall biogenesis [*Pseudomonas fluorescen*s PfO-1]
C2459-Contig1		5.00E-12	potential cell wall glycosidase [*Candida albicans *SC5314]
C2572-Contig1		1.00E-120	cell wall biogenesis protein phosphatase Ssd1, putative [*Aspergillus fumigatus *Af293]
C2766-Contig1		2.00E-24	COG3179: Predicted chitinase [*Pseudomonas fluorescens *PfO-1]
C2907-Contig1		1.00E-51	related to ECM4 protein (involved in cell wall biogenesis and architecture) [*Neurospora crassa*]
C3226-Contig1		0	glucan synthase [*Coccidioides posadasii*]
C3315-Contig1		1.00E-19	1,3-beta-glucanosyltransferase, putative [*Aspergillus fumigatus *Af293]
C3319-Contig1		3.00E-91	chitin synthase class VI [*Coccidioides immitis*]
C3366-Contig1		2.00E-88	cell wall glucanase, putative [*Aspergillus fumigatus *Af293]
C3375-Contig1		2.00E-68	1,3-beta-glucanosyltransferase Bgt1 [*Aspergillus fumigatus *Af293]
C3486-Contig1		1.00E-156	glucanosyltransferase GEL-1 [*Coccidioides posadasii*]
C3510-Contig1		3.00E-12	GPI anchored cell wall protein, putative [*Aspergillus fumigatus *Af293]
EST000306	DW405885	3.00E-44	1,3-beta-glucanosyltransferase Gel2 [*Aspergillus fumigatus *Af293]
EST001651	DW407230	2.00E-17	chitin synthase activator (Chs3), putative [*Aspergillus fumigatus *Af293]
EST001838	DW678357	3.00E-16	cell wall synthesis protein [*Penicillium chrysogenum*]
EST001871	DW678390	4.00E-98	chitin synthase V [*Coccidioides immitis*]
EST002199	DW678718	5.00E-16	beta-1,6 glucan synthetase (Kre6), putative [*Aspergillus fumigatus *Af293]
EST002677	DW679196	9.00E-11	cell wall synthesis protein [*Penicillium chrysogenum*]
EST003205	DW679724	2.00E-07	chitin deacetylase [*Colletotrichum lindemuthianum*]
EST003442	DW679961	6.00E-68	1,4-alpha-glucan branching enzyme [*Aspergillus fumigatus *Af293]
EST003782	DW680301	5.00E-28	cell wall protein (PhiA), putative [*Aspergillus fumigatus *Af293]
EST003793	DW680312	4.00E-06	related to chitinase 3 precursor protein [*Neurospora crassa*]
EST005840	DW682359	1.00E-39	vegetative cell wall protein gp1 [*Aspergillus fumigatus *Af293]
EST005855	DW682374	2.00E-21	class V chitin synthase [*Coccidioides posadasii*]
EST006570	DW683089	7.00E-48	chitinase 6 [*Coccidioides immitis*]
EST006839	DW683358	3.00E-59	beta-1,6 glucan synthetase (Kre6), putative [*Aspergillus fumigatus *Af293]
EST006926	DW683445	1.00E-26	chitinase 3 [*Coccidioides immitis*]
EST009330	DW685849	2.00E-33	chitin synthase activator (Chs3), putative [*Aspergillus fumigatus *Af293]
EST011701	DW688220	4.00E-35	exo-beta-1,3-glucanase (Exg1), putative [*Aspergillus fumigatus *Af293]
EST012488	DW689007	7.00E-10	class V chitinase, putative [*Aspergillus fumigatus *Af293]
EST013666	DW690185	1.00E-37	chitinase [Aspergillus fumigatus]
EST013760	DW690279	1.00E-14	cell wall glucanase, putative [*Aspergillus fumigatus *Af293]
EST015346	DW691865	4.00E-13	Glucoamylase precursor (Glucan 1,4-alpha-glucosidase) (1,4-alpha-D-glucan glucohydrolase)
EST017058	DW693577	9.00E-15	COG0463: Glycosyltransferases involved in cell wall biogenesis [*Anabaena variabilis *ATCC 29413]
EST017163	DW693682	7.00E-42	class V chitin synthase [*Coccidioides posadasii*]
EST018650	DW695169	1.00E-64	glucan synthase [*Coccidioides posadasii*]
EST018654	DW695173	2.00E-14	class III chitin synthase [*Coccidioides posadasii*]
EST018682	DW695201	2.00E-09	cell wall biogenesis protein phosphatase Ssd1, putative [*Aspergillus fumigatus *Af293]
EST019188	DW695707	2.00E-39	exo-beta-1,3-glucanase, putative [*Aspergillus fumigatus *Af293]
EST019502	DW696021	5.00E-21	chitin biosynthesis protein (Chs7), putative [*Aspergillus fumigatus *Af293]
EST019827	DW696346	7.00E-55	chitinase 6 [*Coccidioides immitis*]
EST019908	DW696427	8.00E-17	chitin synthase activator (Chs3), putative [*Aspergillus fumigatus *Af293]
EST019948	DW696467	5.00E-11	related to glucan 1, 4-alpha-glucosidase [*Neurospora crassa*]
EST021575	DW698094	2.00E-60	1,4-alpha-glucan branching enzyme [*Aspergillus fumigatus *Af293]
EST021606	DW698125	1.00E-31	endo-1,3(4)-beta-glucanase, putative [*Aspergillus fumigatus *Af293]
EST022805	DW699324	1.00E-07	glucan synthase [*Paracoccidioides brasiliensis*]
EST023134	DW699653	5.00E-13	beta-1,3-glucanosyltransferase 3 [*Paracoccidioides brasiliensis*]
EST024342	DW700861	1.00E-110	COG0296: 1,4-alpha-glucan branching enzyme [*Pseudomonas fluorescens *PfO-1]
EST024896	DW701415	2.00E-27	1,4-alpha-glucan branching enzyme [*Xanthomonas campestris *pv. campestris str. ATCC 33913]
EST025124	DW701643	5.00E-62	COG0463: Glycosyltransferases involved in cell wall biogenesis [*Microbulbifer degradans *2–40]
EST027343	DW703862	4.00E-17	endoglucanase, putative [*Aspergillus fumigatus *Af293]
EST027819	DW704338	8.00E-14	GPI anchored cell wall protein, putative [*Aspergillus fumigatus *Af293]
EST028103	DW704622	1.00E-55	glucan synthase [*Coccidioides posadasii*]
EST029640	DW706159	1.00E-60	class I chitin synthase [*Coccidioides posadasii*]
EST031669	DW708188	1.00E-60	chitinase [*Aspergillus nidulans*]
EST033214	DW709733	4.00E-87	related to beta-1, 3-glucan binding protein [*Neurospora crassa*]
EST033337	DW709856	6.00E-81	class V chitinase, putative [*Aspergillus fumigatus *Af293]
EST033834	DW710353	7.00E-81	class V chitinase, putative [*Aspergillus fumigatus *Af293]
